# Strengthening Data Systems and Use for Infectious Outbreak Preparedness in Uganda

**DOI:** 10.1007/s44197-026-00602-2

**Published:** 2026-07-02

**Authors:** Ildephonse Nizeyimana, Alex Riolexus Ario, Thumbi Mwangi, Ann Muthiru, Michael Arthur Ofori, Olufemi Adedokun, Ann Mwangi

**Affiliations:** 1 School of Science and Aerospace Studies, Moi University, Eldoret, Kenya; 2 Uganda Public Health Fellowship Program, Kampala, Uganda; 3 Center for Epidemiological Modelling and Analysis, University of Nairobi, Nairobi, Kenya; 4 Department of Anthropology, Gender and African Studies, University of Nairobi, Nairobi, Kenya; 5 University of Cape Coast, Cape Coast, Ghana; 6 University of Ibadan Research Foundation, Ibadan, Nigeria

**Keywords:** Infectious disease outbreaks, Ebola, Health data, Data systems interoperability, Pandemic preparedness, Public health emergencies, Disease surveillance

## Abstract

**Background:**

The Covid-19 pandemic highlighted a critical lesson for global health systems: effective preparedness depends on timely access to reliable and interconnected health data. Across many countries, fragmented information systems limited the rapid exchange and use of data needed for outbreak detection, response, and decision-making. In Uganda, the recurrent emergence of infectious disease outbreaks, including Ebola, Marburg virus disease, and cholera, has reinforced the importance of resilient and interoperable health information systems. Although substantial progress has been made in strengthening disease surveillance and response mechanisms, persistent challenges continue to affect the country’s capacity to generate and utilize high-quality data during public health emergencies.

**Methods:**

This study combined a review of published and grey literature with qualitative inquiry to explore existing challenges in Uganda’s health data ecosystem. Six key informants with expertise in health information systems, disease surveillance, and outbreak response participated in in-depth interviews to provide insights into current gaps and potential solutions.

**Results:**

The findings revealed several interconnected challenges, including fragmented and poorly integrated information systems, limited access to critical digital infrastructures, incomplete digitalization of data collection processes, and weaknesses in data quality and accessibility. These challenges often result in delays in data sharing and evidence-based decision-making during outbreaks. Participants noted that many of these barriers are well recognized and can be addressed through targeted and relatively modest investments. Key recommendations included accelerating the integration of existing health information platforms, expanding end-to-end digital data collection, increasing investments in health information systems, and strengthening coordination among government agencies and development partners.

**Conclusions:**

Strengthening Uganda’s preparedness for future epidemics requires sustained commitment to interoperable health information systems and high-quality data management. Building on the strong foundations already established through collaboration with partners such as World Health Organization and Africa Centres for Disease Control and Prevention, targeted investments and enhanced governance mechanisms could address the most pressing system gaps. Advancing these efforts will improve the availability and use of quality health data, enabling more effective surveillance, faster outbreak response, and greater resilience against future public health threats.

**Supplementary Information:**

The online version contains supplementary material available at 10.1007/s44197-026-00602-2.

## Introduction

### Background on Global Pandemic Preparedness

As the world continues to face various public health threats, many of which are related to climate change, strengthening health infrastructures has been among key priorities. Among them, health data systems need to be reconsidered as the current digital era has made data a strategic resource for decision-making. While diseases are unavoidable, some are preventable and when they occur, they can be effectively controlled when health systems in place are strong. Special efforts are required for the control of infectious outbreaks and this is more dangerous in the current highly interconnected world.

Throughout history, infectious diseases outbreaks have occurred repeatedly, often causing significant loss of life due to inadequate preparedness. For instance, the bubonic plague ($14^{th}$ century), the flu ($20^{th}$ century) and HIV/AIDS (20$^{th}$ and 21$^{st}$ century) are some of the most well-known pandemics in previous centuries [26]. Covid-19 revealed to the world that we are collectively unprepared for any public health threat [9, 20]. The consequences of unpreparedness are not only in the heavy burden imposed and life loss, but also in the perturbation of other health services where every effort is concentrated on the present threat, and more regretfully, with inefficiencies, due to insufficient information. For instance, [23] demonstrated that Covid-19 disrupted various immunization services in all 170 countries and territories, with a notable example being the global reduction in doses of diphtheria–pertussis–tetanus-containing vaccine (DTP3) by 33*%*.

As the global population is getting highly connected, risks are higher and hence, a sustainable and effectively coordinated global response may prevent the exponential spread of such diseases. In [21], they proposed the development of scientific innovations to deal with infectious diseases with pandemic potentials and the strengthening of regional and national initiatives about health development and research, and manufacturing of drug and vaccine. The commitment at all levels including local, national and international level is key. While different experts shared their views on how general pandemic preparedness and response efforts may be guided by lessons learnt from previous pandemics, including Covid-19 [9], those lessons may serve as a reference but are less effective than studies purposively designed to generate preparedness-relevant evidence without being under pressure of the situation at the time. Similarly, in [20], they recommended sustainable and resilient laboratory systems where countries were called to institutionalize knowledge and resources for enhanced collection and analysis of data. Some pathogens such as coronavirus were found to have high pandemic potential, thus highlighting the importance of early planning [26]. [25] proposed an International Pandemic Financing Facility targeting pandemic preparedness through regional and global institutions while [14] suggested a binding treaty and effective resources sharing, but again highlighted the responsibility of countries as crucial. Wealthier countries were said not to share a few means and resources that were available at the rise of covid-19, but that behavior was an obvious and unavoidable reflex in such case of emergency. Shared collective efforts towards preparedness are the best way for equitable resources sharing in case of a pandemic threat because countries’ contributions may depend on their economies.

The pressure of Covid-19 might have awakened us, and the pandemic was so dangerous not only because it was that disastrous, but also unexpected. Many countries were in complete despair, just unaware of what to do. It showed how remaining prepared against any type of disease may ease the burden and stress while dealing with it. Pandemic preparedness is now of key importance for any future public health threat, and the role of effective health data infrastructures may not be downplayed. Countries need to shift away from reactive decision-making where they just decide according to the present pressure, and move to the institutionalized informed-policies. The undeniable role of data in health emergency responses underscores the urgency for strong and sustainable health data infrastructures.

When a region or a country experiences an infectious disease outbreak, coordinated national efforts, in collaboration with WHO and other health partners, can lead to a better response. This has happened in various periods of time where deadly outbreaks were successfully contained. For instance, International Coordinating Group (ICG) on Vaccine Provision established Ebola vaccine stockpile in 2021 from which Uganda got 23,460 vaccines (16*%*), being the second beneficiary after DRC (111,000) and followed by Guinea-Bissau (11,170) [13].

### Preparedness for Infectious Outbreaks in Uganda

#### Overview

Effective improvement processes are grounded in lessons drawn from prior experience. Uganda’s successful containment of the 2022 Ebola outbreak benefited from lessons learned during the COVID-19 pandemic, as well as experiences from the Democratic Republic of the Congo’s response to Ebola Virus Disease (EVD) outbreaks [15]. While strengthening health infrastructure is widely recognized as a key component of global pandemic preparedness, this need is particularly urgent in Uganda, where recurrent outbreaks of Ebola and other infectious diseases continue to pose significant public health challenges.

Failures in data and specimen sharing have been identified as potential barriers to outbreak response research (ORR). To address these challenges, strategies such as ensuring local ownership of data, improving access mechanisms, and reducing legal, ethical, and logistical barriers have been recommended [8]. Effective data access and sharing, both within and between countries, have also been recognized as essential for managing current and future pandemics, although concerns regarding privacy and confidentiality remain important considerations [7]. Similar lessons emerged from several Covid-19 case studies, where improved data sharing and utilization were recommended as important contributors to long-term societal benefit [3].

Despite the recognized importance of data for evidence-informed policymaking, numerous challenges continue to limit its effective use. At the data collection stage, inconsistencies in practices and standards across institutions create barriers to standardization and governance. Given Uganda’s history of recurrent Ebola outbreaks, greater use of data generated from previous outbreaks could substantially strengthen preparedness efforts and improve the prevention and control of future epidemics.

#### Policy Problem

Although "pandemic preparedness" has become one of the most frequently cited lessons from the Covid-19 pandemic, momentum toward preparedness has slowed in many settings following the acute phase of the crisis. Governments and institutions that mobilized rapidly during the pandemic have, in some cases, shifted their attention to competing priorities. The reactive nature of many Covid-19 responses was widely criticized [20], yet it often represented the only available option in the face of limited preparedness. Health data systems were consistently identified as a major weakness during the pandemic because effective response strategies depend on timely, reliable, and evidence-based information. Strong health data infrastructures are not only essential during public health emergencies but are equally important for routine health service delivery and surveillance activities. As such, investments in health information systems should be viewed both as everyday operational necessities and as critical components of pandemic preparedness.

Despite widespread recognition of these lessons, progress toward preparedness remains uneven, particularly across Africa. Developing and implementing a shared preparedness agenda is challenging because countries operate within diverse cultural, social, economic, and political contexts [2]. Consequently, some countries may allocate limited attention and resources to preparedness while addressing other pressing national concerns. This does not necessarily reflect negligence; rather, it often results from competing priorities and resource constraints, although stronger political commitment remains necessary [22]. Furthermore, the global response to the H1N1 influenza pandemic was criticized by some observers as being overly alarmist, leading to skepticism and reducing the translation of lessons learned into practical preparedness measures. Combined with pandemic fatigue and budget reductions, this may have contributed to the widespread lack of preparedness revealed during the Covid-19 pandemic [6].

Despite Uganda’s strong outbreak response mechanisms and its rapid implementation of emergency measures, which have been commended by the WHO, important gaps remain within the country’s health data systems. These gaps continue to limit the effectiveness of outbreak detection, response, and control efforts. Notable achievements include the establishment of the Public Health Emergency Operations Centre (PHEOC) and the National Task Force (NTF) for public health emergencies, both designed to strengthen Ebola preparedness and response [1]. The NTF operates through seven functional pillars that have been decentralized across multiple administrative and geographical levels [18, 24].

#### Current Situation

Uganda became the second country after Bangladesh to deploy the Go.Data tool [27]. Health Information Systems Programme (HISP) Uganda has played a central role in supporting data sharing and utilization during outbreak responses. Developed through collaboration between the Global Outbreak Alert and Response Network (GOARN), the WHO, and other partners, Go.Data has proven valuable for real-time digital data collection and outbreak management. Following the Covid-19 pandemic, approximately 12% of countries expanded their use of Go.Data to cover other disease outbreaks [12]. Uganda has used the District Health Information Software (DHIS) since 2011 through the HISP global network. Following the adoption of the revised Integrated Disease Surveillance and Response (IDSR) guidelines in 2019, the country developed an electronic Integrated Disease Surveillance and Response (eIDSR) system through customization of DHIS2, the latest version of the platform.

The electronic Integrated Disease Surveillance and Reporting for Case-Based Disease Surveillance (eIDSR-CBS) was piloted in the West Nile region between August 2018 and February 2019 [5]. Subsequent upgrades expanded its functionality, and since October 2020, the system has been implemented in 100 of Uganda’s 146 districts [17]. A complementary compendium was also established within the system to document retrospective information on public health emergency responses that have occurred in Uganda since 2000 [4]. In addition, [24] highlighted the effectiveness of the viral haemorrhagic fever surveillance and laboratory programme at the Uganda Virus Research Institute (UVRI), which has played a critical role in the early detection of several infectious diseases.

## Methods

To generate evidence for policy recommendations, we conducted a comprehensive review of published and grey literature on health data systems in Uganda and globally, with particular attention to their role in pandemic preparedness and the management of infectious disease outbreaks. The review helped identify existing gaps, challenges, and best practices related to data collection, management, sharing, and use during public health emergencies.

To complement the literature review and obtain context-specific insights, we conducted in-depth interviews with six key informants (KIs) in Uganda whose professional responsibilities are directly related to health information systems, data governance, disease surveillance, and outbreak response. Key informants were selected through purposive sampling from members of Uganda’s National Task Force (NTF) for Public Health Emergencies. This group was chosen because outbreak response coordination occurs primarily at the national level, where critical decisions regarding data management, analysis, and policy implementation are made. Data generated during outbreaks are routinely channelled to the Ministry of Health for consolidation, analysis, and decision-making.

Following discussions on the study objectives and information needs, our focal team at the Ministry of Health advised that interviews should focus on national-level professionals with direct involvement in outbreak data management and governance. Participants were drawn from both government institutions and partner organizations supporting public health emergency preparedness and response in Uganda. Of the seven individuals identified as relevant to the study, six participated in the interviews. One potential participant was unavailable during the two-week data collection period.

The interview guide was designed to explore the key challenges identified through the literature review and to elicit recommendations for strengthening health data systems. Study information and interview topics were shared with participants at least one week before the interviews. All interviews were conducted virtually via Microsoft Teams at times convenient for the participants. The first interview took place on $31^{st}$ October 2025, while the final interview was completed on $6^{th}$ November 2025.

With verbal informed consent obtained from each participant, interviews were audio-recorded to ensure accurate documentation and analysis. Participants also granted permission for their perspectives to be incorporated into this publication. Each interview involved the interviewer (the first author), the participant, and a note-taker from the SFA Foundation, who was responsible for recording and documenting the discussion.

Interview recordings were transcribed and analysed using thematic analysis. Emerging themes were identified, coded, and organized according to the study objectives. To protect participants’ confidentiality, they were assigned anonymous identifiers (R01–R06), which are used when quoting their views in this manuscript.

## Results: Identified Gaps

Decision-making during an outbreak emergency needs to be timely. A delay of hours can result in more infections, severity of cases and finally deaths. All the gaps mentioned below are constraints to the real-time data collection, data analysis, reporting, and decision-making.

### Gap 1: Unintegrated Systems

Prior studies have highlighted challenges resulting from non-harmonized digital tools [19]. While they commended the digital technology used during Covid-19 in Uganda, the coordination for a harmonized central system may lead to a higher success. This hinders data collation and causes delays in communication and decision-making because of the substantial efforts to retrieve reliable information from different systems, lacking interoperability. This was echoed by R04 in these words: “*My reports delay because I have to ask someone to manually extract data from one system and upload it into another. If the systems were integrated, I wouldn’t need to do that*”.

In general, all six key informants agreed that this specific gap exists, where five of them expressed strong agreement. One respondent (R05) mentioned the governance or organization as being the main issue for many of the gaps starting from unintegrated systems and said: “*The interoperability is the challenge. It is doable, but we just need organization.... There is a need to have inventory of different systems in the play and try to integrate them. It’s not a technology issue, it is a governance issue. So you need to have that guidance and governance*”.

Fortunately, as a widely recognized and pressing challenge, respondents said that there was work in progress towards the solution. For instance, R01 said: “*We’ve tried to put together what we call a National Health data warehouse where we have data in one place, and we’ve developed what we call an Integrated Outbreak Response System. It’s not yet complete, but we really think that it should be able to solve that problem*.” This same respondent said that unintegrated systems make the work harder in these words: “*It affects the data use. You extract data from eIDSR and from Go.Data, you start finding discrepancies in data, and that inconsistency brings delays in investigation. We had a situation where in case management system it’s telling us, for example, we have 50 cases. You go to the lab; they’re telling you that you must be having 65 people, in contact tracing; they’re telling you they have 64. It becomes a complete mess tracking that and reconciling it because of the data sources*". The progress towards integration mentioned by R01 was also confirmed by R03, saying: “*eIDSR was to an extent integrated with Go.data, but we have not been able to use it in a real response. But that integration is there, and it has been tested and it can be set up*”.

Once the systems are already integrated, even for a new outbreak, only a simple readjustment will be needed. R06 said: “*For each of the response pillars, we have standardized tools. When Mpox had just come, it was the first time Uganda was responding to it, so there was need to readjust the tools*”. So, the success in the integration of systems in place would make the task smoother from then onwards.

### Gap 2: Poor Access to Technology Infrastructures

The digitalization requires the availability of certain infrastructure. These include computer hardwares or smart-phones, Internet connectivity and electricity. Even some existing non-interoperable systems were inaccessible in certain geographical locations. Limited access to electricity and the internet made some employed technologies less productive. Especially for Go.Data, poor or lack of internet connectivity resulted in delay in updating datasets. During Covid-19 pandemic, by June 2020, only 56% of all Ugandan health facilities were able to submit daily reports. The 44% that failed to, were due to the lack of internet or its poor networks in the areas. The same issue led to delayed tracking of geographical spread of Ebola outbreak because of difficulties in capturing geographical coordinates [16, 17, 19].

When asked about technology infrastructures, R05 replied: “*Those challenges are there in terms of Internet connectivity. The internet data in the country is not that cheap. You will also find places where you will not be able to have internet data. But the biggest challenge is the governance gap*”. This respondent mentioned the governance gap recalling the management of tools like phones and tablets that were made available during Covid-19 outbreaks but after the outbreak no custodian or storage place were defined that one could find them again. Meanwhile, financial constraints were mentioned to lead to this gap in digital tools where R02 said: “*... funding needs to be made available for us to quickly move in and support response, and procure the digital tools in good time*”.

R06 talked about an arrangement that helped sometimes: “*If we have to buy a phone for everyone, then we’ll lose it. Secondly, they are very easy to personalize. So, our approach in Uganda is that if I am a health worker and have a phone, then the Ministry of Health comes up with a tool and installs it on my phone. The best they can do is to assist me with the data. If we had to make sure that everyone has a gadget before they report, that is a little more expensive*”. In addition to financial constraints to acquire all needed technological infrastructures, even those already available need vigilant coordination. Meanwhile, after identifying internet connectivity and gadgets as challenges, R04 emphasized the coordination issue on the management of tools distributed for data collection where their recovery was reported to be difficult.

### Gap 3: Incomplete Digitalization of the Data Collection Process

Five of the respondents reported that the data collection process is a mix in which some tasks are done on papers and others digitally. One said that the process is completely manual (paper-based), and all of them reported it to be time-consuming because paper-based data require to be entered into a determined system before analysis. The respondent R03 said: “*... when a particular district is declared that it has a suspect or a confirmed case, there’s always a lot of papers that will be there, take about a day or two, depending on how quickly they are deployed. We will start to digitize certain parts ... sometimes it can even take about a week to digitize and sometimes even the whole entire time of the response. There will be a mixture of both the paper and the digital*”. R05 said: “*The health workers usually just give you primary data and they know how to collect it using the paper tools*”.

R01 mentioned a challenge to manage the data collection devices, saying: “*At the community level, the biggest challenge has been device management. When we give these community health workers phones, you know, they’re either breaking them, losing them, putting them up for loans to get money. The only way to solve it is for us to make custom orders from the factory so that the laser engraves the phones and the phones are installed with an MDM [Mobile Device Management] to be able to track them*”. However, this same respondent appreciated how the digitalization makes the work easier, especially with the Ugandan centralized data systems, and said: “*They normally go and set up an Internet connection and give devices/phones for contact tracing. Data is entered by the community, the VHT [Village Health Teams], they enter the data in Go.Data and it comes direct to the centre*”. This shows how the tasks might be easier if the process were completely digital. It is worth noting that this challenge of management reported by R01 supports the idea of R04 in Gap 2 above. Finally, one may understand that Gap 3 results from Gap 2.

### Gap 4: Poor Quality Data

This emerges as a result of the three previous gaps. Datasets from different tools are inconsistent and incomplete. In some cases, data cleaning has been a burdensome task, in some cases requiring additional staff to be hired, which consequently delayed the availability of real-time data [16, 19]. In addition to the missing data confirmed by all the six respondents, timeliness was also a challenge but said to be sometimes avoided by the fact that anyone will submit whatever data they obtain since the situation was declared an emergency. With the unintegrated systems, inconsistencies become unavoidable as explained by respondent R01 in Gap 1. For instance, when asked about the main problems about data qualities, R02 said: “*Mainly it [the issue] is having incomplete data. For example, you’re reporting about the case and maybe the age is missing because maybe the person did not interrogate well to get the age or some aspect*". This respondent mentioned about the delays also and this was supplemented by R04 who said: “*Some reports delay because some data are manually pulled from a system to be uploaded in another. The problem with data quality is mostly missing variables*”. This cannot be fully attributed to fragmented systems alone, but also to the emergency environment, pushing data collectors to undervalue some variables. R04 continued in these words: “*All those fields are missed out because they [data collectors] are rushing to just complete demographics and then symptoms and that’s it. Most of them feel convenient with the demographics, age, sex, village and then a few symptoms*”. R06 mentioned logistical challenges to contribute to the incompleteness of data where they may fail to seem as if hunting some suspects who behave as hiding. So, with some possibility to achieve the desired timeliness, incompleteness and inconsistencies in data remain unavoidable during outbreaks.

### Gap 5: Insufficiency of Specialized Personnel

As a new digital tool is introduced, some health facilities may struggle to find skilled personnel to use the introduced tools. While some trainings were done and cascaded to lower levels from national level, some health workers find it burdensome to be responsible for data collection in addition to their usual workload. If there were data managers specifically responsible for this task, the process might be smoother [17]. This was reaffirmed by R05 who said: “*The only challenge would be when there is too much work. There are few health workers and quite a lot to be done*”. When asked about the training for personnel, the same respondent replied: “*I think it should be improved in the sense of not training only during the outbreak. It should be continuous, maybe annually. It’s done only during emergencies*”. The arrangement of using their own phones which was mentioned in Gap 2 was also described as an addition to the increased workload where R03 said: “*There’s a need to do training. We need to make sure that they understand the tools that they’re using and are using them as they should. The biggest challenge is usually extra workload, sometimes having to use their own devices. Sometimes having to even use their own Internet data just to be able to submit*". The same respondent said that as they aim at integrating systems in place, they should also continue to build a community of people who can use these tools.

Meanwhile, in presence of an outbreak, the country manages to get a way out despite the lack or insufficiency of trainings in normal times. The respondent R02 described the situation as follows: “*The skills are lacking. Before the outbreak comes, they will have no skills to do with that. Then when it comes, we will move in and train them on how to use this phone and this application too*”. When asked about the frequency of trainings given, the same respondent continued: “*Even when we wanted to train as we had planned once a quarter, we may not be able to do that because of limited funding. But before we used to try. So yes, the trainings’ frequency is not as we had planned*”. There is a need for regular trainings to remain assured of a workforce able to respond to a wide outbreak.

### Notice

(1) The lack of clear guidelines governing outbreaks’ data sharing to some stakeholders, and especially academic researchers who should effectively use the data in their studies had been mentioned as another challenge in previous literature [16, 19]. However, 4 out of the 6 respondents disagreed with this, where for example, R04 said: “*It’s just probably that the guidance has not been disseminated, but if there is any information you’re seeking from the health sector, including outbreak related data, you seek for permission through the Director General Health Services*”. R01 also categorically rejected that claim saying: “*If right now you sit on your computer and search for Uganda Health data access and use guidelines, the guidelines are very clear. If you need any data set for any purpose, write officially to the Ministry of Health Director General*”. So, this is no longer an issue, but it probably used to be an issue some years back.

Moreover, in [11], they had identified challenges in the coordination of stakeholders. It was confirmed by R05 who said: “*Yes, development partners have been coming in with their own tools. That’s true and that’s the reason why we have very many systems and they are not interoperable because every stakeholder comes with their own tools because they think what is probably available is not comprehensive.... you find three different stakeholders are doing the same thing using different tools*”. However, R02 affirmed that this issue has been sorted out, saying: “*We wouldn’t like the partners to really come and take leadership of all these systems. Theirs is to come and complement what we have*”. Another achievement is that from the time of Covid-19, all outbreaks-related data are shared in one lab information system repository, which makes the data available.

(2) Regardless the challenges identified, Uganda is among a very small number of Sub-Saharan countries that are on good track in terms of infectious outbreaks’ preparedness, especially taking into account data systems. This is also confirmed by R03 who said: “*I think we are on the right path and we’ll continue to improve and we’re always better prepared whenever we have another outbreak because of building on top of what we started with in the previous*”.

## Discussion

### Main Findings

Many challenges in public health infrastructures need to be treated with due attention. More specifically, health data infrastructures are useful in daily routine health data collection and management. During an outbreak, they also become an important asset in the surveillance and control of those threats. Meanwhile, inadequacy of health systems is a general concern, especially in low- and middle-income countries. The sub-Saharan Africa which bears a heavy burden of various infectious and non-communicable diseases needs to make more efforts. In general, Uganda is among sub-Saharan countries which are performing relatively well in terms of outbreak preparedness, where it is ranked $63^{rd}$ of 195 countries globally, with a score above both African and global averages [10]. This awareness resulted from the recurrence of infectious outbreaks among which some were harshly deadly. Besides the Democratic Republic of Congo and Malawi that also learned from nearly similar experiences, even Sierra Leone and Liberia were reported to make remarkable efforts after the deadly West Africa Ebola outbreak in 2014 [2]. So, repeated exposure to such threats has helped countries gradually improve their responses.

Contrarily to Uganda, there may be countries that made minimal further improvement after Covid-19. The latter obviously awakened many countries as the most recent global public health threat. As mentioned above, countries like Uganda which stay in recurrent fights against various outbreaks are relatively better prepared. After Covid-19, some other countries may feel that the pressure has eased, leading to the probable deceleration of preparedness measures. So, despite the gaps identified in Uganda, there is likelihood that those other countries are still relatively behind. Such countries should learn from Uganda although Uganda itself still has room for improvement. This should serve as a particular call to action for many African countries known to have fragile health systems.

Funding for outbreak preparedness needs to be improved from both national governments and international organs. Since there is no guarantee that the World may not meet a pandemic similar to Covid-19 or with higher intensity, collective efforts for outbreak preparedness should lead to a better success in the future. While the data systems should be strengthened with aim of better response to future outbreaks, once present, they may also be useful even in data collection and management for other diseases. More especially, since Africa carry nearly 50% of the burden from many diseases, it is not justifiable that the same continent remains data-poor in terms of health information. Being aware of the value of data in this current digital era, it should be an urgent call for the African continent to collectively invest in health data systems. Moreover, the current shift towards precision medicine is accelerated by the availability of data, which Africa should have in abundance, if adequate data infrastructure and measures were in place.

It is concerning that the same challenges have persisted for two decades. While there have been gradual improvements as they learned from each outbreak, the rate of learning has probably not been sufficient. There may also have been a lack of rigorous policies or suboptimal prioritization. Although Uganda was hit by the first Ebola outbreak in 2000, it is more likely that the awareness didn’t rise in the first instances. With recurrent outbreaks, this was probably when the attention towards infectious outbreaks preparedness was drawn. Currently, being a global concern, there is hope for accelerated and collective efforts towards global pandemic preparedness.

According to one respondent, strengthened governance remains the key prerequisite for effectively addressing all identified gaps. If the ongoing systems integration remains a consistent priority, it could potentially be achieved in the relatively near term, through an already-existing partnership with Uganda HISP, with no substantial additional budgetary resources. Once secured, an estimated budget of $500,000 can solve, to a certain extent, the constraint of electronic gadgets (phones or tablets) for the digitalization of data collection process. With this amount, around 1500 gadgets can be reserved for use in case of an outbreak. A telecommunication company chosen as a partner can assist making the gadgets trackable for their safety as also suggested by one respondent (R01). An online platform could be built to help youth volunteers train themselves through a self-paced course to use the standardized tool when completed. With a budget of around $30,000, this could be initiated and would address the skills and personnel gap at some level. This can help the country have a standby workforce in case of a wider outbreak requiring additional workers or to replace trained employees who might have moved away. The option of self-paced course will also help to address, to some extent, the challenge of trainings which have been reported not to be regular due to budget constraints. The Ministry of Health should assume the coordination role in the already-existing and active partnerships between Uganda and WHO, Africa CDC, Uganda HISP among other partners.

### Strengths and Limitations of the Study

The major strength of this work resides in its relevance to health policy, and especially to pandemic preparedness. The key informants were in good position to know gaps in data systems in Uganda. Therefore, their views are reliable. The data used were collected primarily for this work, reflecting the ground truth. The findings can be expanded in other resource-constrained settings, nearly common in sub-Saharan Africa.

On the other hand, this study got some limitations especially in terms of a small sample size. Despite the relevance of interviewed key informants, one could get richer and more insights if the sample were larger. Especially field data collectors would provide more clarity on challenges met. For instance, it was reported that all variables on the questionnaires are not considered while collecting data. However, in addition to the fact that the objectives outlined in the scope of our study were successfully accomplished, we could not get time and funds that might allow us to go further. Otherwise, there is an extent to which this matter can be explored to more insights from a larger pool of respondents from different levels of responsibilities. Moreover, both the interviewees and interviewer used English during their conversations while it is not their first language. This might affect the depth of ideas for some. Again, even the engagement during online conversations can be limited in one way or another.

## Policy Recommendations

According to the literature review and insights from the in-depth interviews with the Key Informants, we obtained five recommendations:

1. **Accelerate the works towards the integration of available systems**: This is the primary solution, which will also address other challenges, at a certain extent. As such, it requires dedicated and intensive effort.
2. **Complete digitalization of data collection tasks**: This will improve time efficiency.
3. **Enhanced coordination and management**: Closer collaboration with telecommunication companies can help in equipping digital tools used in data collection with internet connectivity for smoother data transmission, and make them trackable for easy recovery. Strengthened accountability and governance mechanisms are needed for improved effectiveness.
4. **Establishment of a standby workforce of volunteers to intervene in case of a wider outbreak**. After the integration of available systems, an online self-paced course for the use of the resulting standardized tool can be set up, and encourage volunteers to learn about it. Those volunteers would serve as a reserve force, just as the course can be a pre-requisite in the recruitment for some positions.
5. **Collaboration with academic researchers**: They will give insights on variables needed to carry out researches that answer various fundamental questions, after responding to an outbreak.

## Supplementary Information

Below is the link to the electronic supplementary material.

Supplementary file 1 (pdf 229 KB)

## Data Availability

The data used and analysed during this study are available from the corresponding author on reasonable request.
