# Supplementary material for: Strengthening Data Systems and Use for Infectious Outbreak Preparedness in Uganda
Source: J Epidemiol Glob Health. 2026 Jul 2;16(1):101. doi: 10.1007/s44197-026-00602-2 (PMC13462018; doi:10.1007/s44197-026-00602-2)
Supplement: Supplementary file 1 [file 44197_2026_602_MOESM1_ESM.pdf]

This survey intends to get insights from infectious outbreaks (like Ebola and others) surveillance team members. The aim is to make a policy brief identifying gaps/challenges especially in data systems and possible recommendations. The output will guide efforts for the effective prevention and response to future infectious outbreaks.

Respondent's identification:

Gender:

Current position:

Role in previous Ebola or any other infectious outbreak response:

1. How many times have you been a member of response team during infectious outbreaks?
    - … times
2. Which infectious disease was it? …….

UNINTEGRATED SYSTEMS

3. How can you describe the data collection process?
    - Digitally entered in database at first hand
    - Manually taken on papers/reports and entered in database after
    - Mixed (paper-based& digitally)
    - Not aware
    - Other (specify)
4. To what extent do existing digital systems (e.g., eIDSR, Go.Data) integrate or share data effectively?

    Very good   Good   Poor   Not sure

What challenges exist in ensuring **interoperability** between systems/tools and institutions?

5. During outbreaks, data managers and data scientists
    - Get data from clinicians' reports and disease registries, and organize them accordingly.
    - Contribute in the data collection protocol design in order to get comprehensive data.
    - Other (specify)

Overall:

Strongly agree   Agree   Neutral   Disagree   Strongly disagree

POOR ACCESS TO TECHNOLOGY INFRASTRUCTURES

6. What are the biggest digital infrastructure bottlenecks? (select all that apply)
    - Server capacity
    - Internet access
    - System downtime
    - Power interruption
    - Health facilities lacking appropriate computers
    - Focal persons lacking smartphones for reporting
    - Other (specify)

7. How adequate is the access to **computers, the internet, and power supply** to support real-time reporting?

Probe:

- Do all health facilities have functioning computers or smartphones?
- How often do you face connectivity or electricity interruptions during outbreaks?

8. Are there standardized tools and formats for data collection across all levels (national, district, community) during outbreaks?
    - Yes (specify)
    - No
    - Not sure

What are the main **data flow pathways** (from health facility to MoH)?

How timely is the feedback loop between field data collection and central decision-making?

9. Are you aware of any decision support system (DSS) that automatically facilitate timely decision-making, during an Ebola outbreak?
    - Yes (specify)
    - No
    - Not sure (any gap for possible improvement?)

Overall:

Strongly agree Agree Neutral Disagree Strongly disagree

POOR QUALITY AND INCOMPREHENSIVE DATA

10. What common data quality issues are often faced during outbreaks? (select all that apply)
    - Missing data
    - Duplication
    - Timeliness (delays)
    - Inconsistencies
    - None
    - Not sure
    - Other(specify) (what is the cause?)

11. Is there any database where one can find data for all past Ebola outbreaks?
    - Yes
    - No
    - Not sure (Do you think it can be useful? How?)
12. Do you think the scope of data collected during Ebola outbreaks is sufficient to inform decision-making and for predictive analytics (e.g.: clinical data, demographic info, risk factors, behavioral data)?
    - Yes, it is comprehensive
    - Partially sufficient, some key areas missing
    - No, major data gaps exist
    - Not sure

Are there sufficient resources (human, technological, and financial) to sustain digital surveillance systems like eIDSR or Go.Data post-outbreak? What can you suggest?

13. Which category of data you think is missing or given little consideration during Ebola outbreaks (that should help in further investigations like individual risk profile, …)? (select all that apply)

    Socio-demographic Data
    - Age, sex, ethnicity, and race
    - Education level and literacy
    - Marital status and family composition
    - Geographic location and residence history

    Socioeconomic data
    - Household size and income
    - Occupation and employment status
    - Health insurance coverage
    - Access to digital tools (phone/internet)
    - Food security status

Biological & Physiological Data

- Vital signs (blood pressure, heart rate, temperature, respiratory rate)
- Laboratory test results (blood work, urine tests, biomarkers)
- Genetic and genomic information
- Anthropometric measurements (height, weight, BMI)
- Organ function tests (kidney, liver, heart function)
- Imaging results (X-rays, CT scans, MRI, ultrasound)

Behavioral & Lifestyle Data

- Dietary habits and nutrition intake
- Physical activity and exercise patterns
- Substance use (tobacco, alcohol, drugs)
- Sleep patterns and quality
- Sexual and reproductive behavior

Clinical & Medical Data

- Medical diagnoses and disease history
- Symptoms and clinical presentations
- Treatment plans and interventions received
- Medication history and current prescriptions
- Allergies and adverse reactions
- Underlying health conditions (e.g., diabetes, hypertension)
- Vaccination history (COVID-19, influenza, etc.)
- Medications currently taken

Environmental or contextual data

- Sanitation and water access
- Local disease outbreaks or environmental risks
- Occupational exposures and workplace conditions
- Residential environment and housing quality
- Travel history and geographic mobility

Overall:

Strongly agree Agree Neutral Disagree Strongly disagree

THE LACK OF GUIDELINES FOR DATA SHARING

14. Are there guidelines/policies and protocols guiding Ebola data collection, sharing and ethical use?
    - Yes (If yes, identify them and provide critiques if any).
    - No
    - Not sure

15. How effective is the use of the Data Protection and Privacy Act (2019) in guiding data governance during outbreaks?

    Very good   Good   Poor   Not sure (Any challenge or gap?)

16. How do you rate the availability/accessibility of Ebola data for academic researchers?
    - Good
    - Fair
    - Poor
    - Not sure

Are there **formal data-sharing agreements or protocols** between MoH, research institutions, and international partners such as the WHO or others?

How smooth is it during vs after outbreak?

17. From your perspective, what **policy reforms or regulatory frameworks** would most improve Ebola data governance and use in Uganda?
18. How can Uganda better **balance ethical data protection with rapid data sharing** for research and outbreak control?

Overall:

Strongly agree   Agree   Neutral   Disagree   Strongly disagree

STAKEHOLDERS' COORDINATION

19. What are the biggest coordination challenges among stakeholders?

    - Inoperable data systems,
    - Differing reporting standards,
    - Data ownership concerns
    - Other (specify)

20. From your perspective, do you think districts' autonomy to analyze their own data would make district-level data collection and use more effective?

- Yes
- No
- Not sure (how/why?)

21. Has data ownership ever been a concern among stakeholders?

Overall:

Strongly agree Agree Neutral Disagree Strongly disagree

INSUFFICIENCY OF SPECIALIZED PERSONNEL/NEED OF TRAINING FREQUENCY

22. Are health workers serving as data collectors well-trained on reporting protocols?
Probe:

- How frequently do they receive training or refresher sessions?
- Do you provide feedback or supervision to help improve their reporting
- Do they have enough knowledge about ethical data use and governance?

23. How would you assess the technical and human capacity in the surveillance team? What about health workers serving as data collectors?

Very good Good Poor Not sure

24. How do you rate training or technical support and follow-ups targeting the strengthening of surveillance and data systems?

Very good Good Poor Not sure

Overall:

Strongly agree Agree Neutral Disagree Strongly disagree

Is there any additional insight or suggestion you would like to share to help strengthen outbreaks' surveillance data ecosystem in Uganda for an effective preparedness and response to (Ebola) outbreaks??
